# CYP2B6 Functional Variability in Drug Metabolism and Exposure Across Populations—Implication for Drug Safety, Dosing, and Individualized Therapy

**DOI:** 10.3389/fgene.2021.692234

**Published:** 2021-07-12

**Authors:** Immaculate M. Langmia, Katja S. Just, Sabrina Yamoune, Jürgen Brockmöller, Collen Masimirembwa, Julia C. Stingl

**Affiliations:** ^1^Institute of Clinical Pharmacology, University Hospital of Rheinisch-Westfälische Technische Hochschule Aachen, Aachen, Germany; ^2^Department of Clinical Pharmacology, University Medical Center Göttingen, Georg-August University, Göttingen, Germany; ^3^African Institute of Biomedical Science and Technology (AiBST), Harare, Zimbabwe

**Keywords:** drug metabolism, adverse drug reaction, cytochrome P450 2B6, genetic polymorphism, drug safety, pharmacogenetics, pharmacogenomics, personalized medicine

## Abstract

Adverse drug reactions (ADRs) are one of the major causes of morbidity and mortality worldwide. It is well-known that individual genetic make-up is one of the causative factors of ADRs. Approximately 14 million single nucleotide polymorphisms (SNPs) are distributed throughout the entire human genome and every patient has a distinct genetic make-up which influences their response to drug therapy. Cytochrome P450 2B6 (CYP2B6) is involved in the metabolism of antiretroviral, antimalarial, anticancer, and antidepressant drugs. These drug classes are commonly in use worldwide and face specific population variability in side effects and dosing. Parts of this variability may be caused by single nucleotide polymorphisms (SNPs) in the *CYP2B6* gene that are associated with altered protein expression and catalytic function. Population variability in the *CYP2B6* gene leads to changes in drug metabolism which may result in adverse drug reactions or therapeutic failure. So far more than 30 non-synonymous variants in *CYP2B6* gene have been reported. The occurrence of these variants show intra and interpopulation variability, thus affecting drug efficacy at individual and population level. Differences in disease conditions and affordability of drug therapy further explain why some individuals or populations are more exposed to CYP2B6 pharmacogenomics associated ADRs than others. Variabilities in drug efficacy associated with the pharmacogenomics of *CYP2B6* have been reported in various populations. The aim of this review is to highlight reports from various ethnicities that emphasize on the relationship between CYP2B6 pharmacogenomics variability and the occurrence of adverse drug reactions. *In vitro* and *in vivo* studies evaluating the catalytic activity of CYP2B6 variants using various substrates will also be discussed. While implementation of pharmacogenomic testing for personalized drug therapy has made big progress, less data on pharmacogenetics of drug safety has been gained in terms of CYP2B6 substrates. Therefore, reviewing the existing evidence on population variability in CYP2B6 and ADR risk profiles suggests that, in addition to other factors, the knowledge on pharmacogenomics of CYP2B6 in patient treatment may be useful for the development of personalized medicine with regards to genotype-based prescription.

## Introduction

Adverse drug reactions (ADRs) are globally one of the major causes of morbidity and mortality (Giardina et al., 2018; Patel and Patel, 2018). According to the World Health Organization (WHO), ADR is a noxious and unintended response to a medication (Agency, 2017). ADRs can range from mild to severe causing ~6.5% of all visits to the emergency department and longer duration of hospitalization (Giardina et al., 2018; Patel and Patel, 2018; Schurig et al., 2018). Drug clearance may vary up to 10-fold between two individuals of the same weight taking the same drug dosage (Stingl et al., 2013). This variation can be influenced by pathophysiological, physiological, or environmental factors. However, genetic polymorphisms in drug transporters, drug targets and most importantly drug metabolizing enzymes have been emphasized over the years as one of the major factors causing variability in drug response (Weinshilboum, 2003; Evans and Relling, 2004).

The cytochrome P450 (CYPs) enzymes are involved in phase I drug metabolism. Variability in patient exposure and response to various medication have been associated to genetic variants in genes that code for CYP enzymes (Lynch and Price, 2007; Zanger and Schwab, 2013). The human genome harbors 18 CYP families divided into 41 subfamilies encoding 57 genes. Specifically, CYP1, CYP2, and CYP3 families catalyze the biotransformation of many xenobiotic agents. *CYP2B6* is the only gene in the human CYP2B subfamily encoding a functional enzyme (Nebert et al., 2013).

The *CYP2B6* gene which consists of nine exons is located on chromosome 19 at position 19q13.2. It is highly expressed in the liver, and to a certain extent in the extrahepatic tissues such as brain, kidney, digestive tract and the lungs (Lonsdale et al., 2013). *CYP2B6* is a polymorphic cytochrome P450 enzyme with many single nucleotide polymorphisms (SNPs) encoding thirty-eight variants. These variants are referred as star alleles on the Pharmacogene Variation website with designated clinical function as normal, decrease, increase, no or uncertain function (Thorn et al., 2010). Compared to other well-studied phase I enzymes such as CYP2D6, CYP2C19 and CYP2C9, CYP2B6 at first had been thought to play a minor role in human drug metabolism (Desta et al., 2021). However, with the increase in techniques to evaluate its regulation, relative hepatic expression and function, it became evident that CYP2B6 constitutes up to 10% of the functional CYP enzymes in the liver. It is involved in the metabolism of 10–12% of all drugs commercially available in the market (Hanna et al., 2000; Rendic, 2002) and accounts for the metabolism of 4% of top 200 drugs in the market (Zanger et al., 2008). Specifically, it is fully or partially involved in the catalytic biotransformation of at least 90 drugs. Table 1 shows selected drug substrates which are metabolism by CYP2B6.

**Table 1:** Drug substrates known for metabolism by the CYP2B6 enzyme.

**Therapeutic class**	**Substrate^a^**	**Reaction (type)^b^**	**Other CYPs^c^**	**References**
Antiretroviral	Efavirenz	8-hydroxylation (major reaction)	CYP3A4, 3A5, 1A2, 2A6	Patel and Patel, 2018
	Nevirapine	Hydroxylation (major reaction)	CYP3A4 3A5, 2C9, 2D6	Giardina et al., 2018
Antimalarial	Artemether	Demethylation (minor reaction)	CYP3A4, 3A5	Agency, 2017
	Artemisinin	Unknown (major reaction)	CYP3A4	Agency, 2017
Anticancer	Cyclophosphamide	4-hydroxylation (major reaction) N-dechloroethylation (minor reaction)	CY2C19, 3A4, 2E1	Stingl et al., 2013; Schurig et al., 2018
	Ifosfamide	4-hydroxylation N-dechloroethylation (major reaction)	CYP3A4, 2C9, 2C19, 2C8	Stingl et al., 2013
	Tamoxifen	4-hydroxylated (minor reaction)	CYP2D6, 3A4, 2C9, 2C19	Weinshilboum, 2003; Evans and Relling, 2004
Antidepressants	Bupropion	Hydroxylation (major reaction)	CYP3A4, CYP2D6,	Lynch and Price, 2007; Zanger and Schwab, 2013
	Esketamine	N-demethylation (major reaction)	CYP3A4, 2C9, 2C19	Lonsdale et al., 2013; Nebert et al., 2013
	Vortioxetine	Hydroxylation (minor reaction)	CYP2D6, 3A4/5, 2C19, 2C9, 2A6, 2C8	Thorn et al., 2010
	Amitriptyline	N-demethylation (minor reaction)	CYP2C9, 2C19, 1A2, 3A4, 2D6, 2B6, 2C8	Desta et al., 2021
	Fluoxetine	N-demethylation (minor reaction)	CYP2D6, 2C9, 3A4	Rendic, 2002
	Mianserin	N-demethylation (minor reaction)	CYP3A4, 2C19, 1A2	Hanna et al., 2000
	Sertraline	N-demethylation (minor reaction)	CYP3A4, 2C19, 2D6, 2C9	Zanger et al., 2008; Sarfo et al., 2014
Anticonvulsants	Phenytoin	Oxidation (minor reaction)	CYP1A2, 2A6, 2C19, 2C8, 2C9, 2D6, 2E1, CYP3A4	Kharasch and Greenblatt, 2019
	S-mephenytoin	N-demethylation (minor reaction)	CYP2C9, 2C19	Chaivichacharn et al., 2020
	Carbamazepine	3-hydroxylation (minor reaction)	CYP3A4, CYP2C8, CYP3A5	Bank et al., 2019
	Valproic acid	4, 5-hydroxylation (minor reaction)	CYP2C9, 2A6, 3A5	Kim et al., 2017
Anxiolytics, anticonvulsants	Diazepam	N-demethylation (minor reaction)	CYP2C19, 3A4, 3A5, 2C9	Bielinski et al., 2014
	Clotiazepam	N-demethylation (minor) reaction	CYP3A4, 2C18, 2C19,	Dolgin, 2011
	Clobazam	N-demethylation (minor reaction)	CYP3A4, 2C19	Drozda et al., 2018
	Temazepam	N-demethylation (minor reaction)	CYP2C19, 3A4	Bielinski et al., 2014
Anesthetics	Kitamine	N-demethylation (major reaction)	CYP3A4, 2C9	Lonsdale et al., 2013
	Propofol	4-hydroxylation (major reaction)	CYP2C9	Hesse et al., 2004; Zanger and Schwab, 2013
Opioid	Methadone	N-demethylation (major reaction)	CYP3A4, 2C19, 2C9, 2C8, 2D6,	Ekins et al., 1998; Lang et al., 2001
MAOI	Selegiline	N-demethylation (major reaction)	CYP2C19, 3A4, 1A2, 2D6, 2C9	Wang et al., 2003a,b
Antiplatelet	Clopidogrel	Oxidation (minor reaction)	CYP1A2, 2B6, 2C19, 2C9, 3A4	Lee et al., 2003
Smoking cessation agent	Nicotine	N-demethylation (minor reaction)	CYP2A6, 3A4	Aitken and Morgan, 2007; Liptrott et al., 2009
Analgesics	Tramadol	N-demethylation (minor reaction)	CYP2D6, 3A4,	Faucette et al., 2004; Lo et al., 2010
	Diclofenac	5-hydroxylation (minor reaction)	CYP2C8, 2C19, 2C9	Pearce et al., 2016
Gastro-intestinals	Loperamide	N-demethylation (minor reaction)	CYP219, 3A4, 2D6, 2C8,	Lewis and Lake, 1997
Steriod	Testosterone	Hydroxylation (minor reaction)	CYP3A4, 2C9, 2C19	Ekins et al., 2008
Anticoagulant	Coumarin	Aromatic hydroxylation (minor reaction)	CYP2A6, 1A1, 1A2, 3A4	Xie et al., 2003
SERM	Ospemifene	Hydroxylation (minor reaction)	CYP3A4, 2C9, 2C19,	Hidestrand et al., 2001

a *According to the drug Bank (https://go.drugbank.com/categories/DBCAT002619) and online literature*.

b *CYP2B6 is either the major enzyme or the minor enzyme in the biotransformation of the drug. The type of metabolic reaction according to the information from Pharmacogenomics Knowledge base (PharmGKB) website, drug bank and online literature*.

c *Other cytochromes that are involve in the metabolism of the drug, taken from PharmGKB website, drug bank and online literature*.

*SERM, Selective estrogen receptor modulators*.

*MAOI, Monoamine oxidase inhibitors*.

Interestingly, variability in the expression and function of the CYP2B6 enzyme alters the metabolism of these substrates leading to altered pharmacokinetics and therapeutic efficacy. Abnormal drug efficacy associated with patient CYP2B6 genotype has been reported in various populations (Sarfo et al., 2014; Kharasch and Greenblatt, 2019; Chaivichacharn et al., 2020). The scope of this review is to report the evidence on ADRs of CYP2B6 substrates and elucidate possible functional mechanisms of the influence of CYP2B6 polymorphisms on enzyme function and ADRs. Population disparity in the use of CYP2B6 substrates and consequent exposure to substrate-related ADRs are discussed. Serious ADRs due to high-risk pharmacogenetic variants might be avoided by the use of preemptive genotyping (Dolgin, 2011; Bielinski et al., 2014; Kim et al., 2017; Bank et al., 2019) and the use of pharmacogenetic testing has greatly improved the lives of many patients (Lonsdale et al., 2013; Drozda et al., 2018). The knowledge on pharmacogenomics of CYP2B6 in patient treatment may be useful for the development of personalized medicine with regards to genotype-based prescriptions.

## Factors That Influence CYP2B6 Expression and Function

A significant interindividual variability in the mRNA expression, protein levels and activity of CYP2B6 has been reported in human liver microsomes (Ekins et al., 1998; Lang et al., 2001; Hesse et al., 2004). This variability is caused by the following factors; transcriptional regulation involving inhibition or induction of *CYP2B6* expression via the constitutive androstane receptor (CAR) and/or pregnane X receptor (PXR) (Wang et al., 2003a), inductive expression via glucocorticoid receptor (GR) (Lee et al., 2003; Wang et al., 2003b), inhibition of *CYP2B6* by cytokines through CAR and PXR (Aitken and Morgan, 2007; Liptrott et al., 2009), induction of *CYP2B6* by estrogen via the estrogen responsive element (ERE) (Faucette et al., 2004; Lo et al., 2010) and most importantly genetic polymorphism in the *CYP2B6* gene itself (Lang et al., 2001). Developmental regulation (age), gender and disease condition are other confounders of *CYP2B6* differential expression and function (Pearce et al., 2016). It is estimated that genetic polymorphisms and/or gene regulation are the major factors that impact variability in *CYP2B6* expression and function.

## Substrates of CYP2B6

Previous investigations revealed diversity in the structure among CYP2B6 substrates (Lewis and Lake, 1997). They also confer differences in the site of metabolism (Lewis and Lake, 1997). Typically substrates of CYP2B6 are hydrophobic small molecules, neutral or weak bases, very lipophilic with one or two hydrogen-bond acceptors (Ekins et al., 2008). Table 1 indicates that CYP2B6 catalyzes demethylation, hydroxylation and oxidation reactions to form active or inactive metabolites (Hidestrand et al., 2001; Xie et al., 2003; Ekins et al., 2008; Zhang et al., 2017). Substrates of CYP2B6 are found in ~23 different therapeutic classes (Table 1). It is predicted that CYP2B6 is the major catalytic enzyme in the biotransformation of important drugs commonly used worldwide. In combination with other cytochromes, CYP2B6 also plays a minor role in the metabolism of other xenobiotics. Notably, common metabolism of drugs is mediated by CYP2B6, CYP2C19, and CYP3A4 in terms of metabolism of clinically relevant therapeutics (Table 1). The CYP2B6 enzyme confers stereoselectivity, showing higher K_M_ for certain enantiomers such as S-efavirenz, S-mephenytoin, S-fluoxetine, S-ifosfamide, S-methadone, S-ketamine, S-bupropion (Coles and Kharasch, 2008; Ekins et al., 2008; Rakhmanina and van den Anker, 2010). CYP2B6 variants are substrate specific in their metabolic function. Thus, evaluating the impact of each enzyme variant on the metabolism of a specific substrate is of clinical importance (Ariyoshi et al., 2011). For example, recombinant CYP2B6^*^6 showed a decreased metabolism for both efavirenz and cyclophosphamide, while CYP2B6^*^4 showed increased metabolic activity toward efavirenz but less efficient metabolic activity toward cyclophosphamide (Ariyoshi et al., 2011).

## CYP2B6 Allele Variability Across Populations

Wide variability in CYP2B6 allelic frequencies is reported across populations. There exists intra and interethnic variability in the frequency of CYP2B6^*^6 and CYP2B6^*^18 variants. For example, at global level, the minor allele frequency of the CYP2B6^*^6 ranged from 0.33 to 0.5 in African Americans and Africans, 0.10–0.21 in Asians, 0.14–0.27 in Caucasians and 0.62 in Papua New Guineans (Zanger and Schwab, 2013; Rajman et al., 2017). Furthermore, allele frequency at the global level may not represent intraethnic differences within a specific population (Table 2). Though the CYP2B6^*^6 allele is more frequent in Africans and people of African descent, large intraethnic variability within these populations is observed (Rajman et al., 2017). For instance, the frequency of CYP2B6^*^6 in the Nigerian Yoruba population was reported to be 42%, Kenya Kikuyu 34% and Tswana of Bostwana 22% (Table 2). Also, the frequency of CYP2B6^*^9 (G516T) which forms part of the CYP2B6^*^6 variants was reported to be 55% in the Congolese, 20% in South African Xhosa and 37% in the Cameroonian population (Table 2). CYP2B6^*^4 is more frequent in the African, American, and Asian compared to the European population (Table 2). Meanwhile CYP2B6^*^2 is more frequent in the European and African compare to the Asian population (Table 2). This differences are associated with CYP2B6 functional variability and the occurrence of substrate specific ADR.

**Table 2:** Selected studies revealing variability in allele frequency of CYP2B6 alleles in different ethnicities.

**Country/region/population**	***N***	CYP2B6*2	CYP2B6*4	CYP2B6*6	CYP2B6*9	**References**
**Europe**						
German	430	5.3	4	32.1	28.6	Giardina et al., 2018; Patel and Patel, 2018
Spaniard	360	-	6.2	21.5	1.4	Agency, 2017
Swiss	226		3.9	24.8	26	Giardina et al., 2018; Schurig et al., 2018
British	270	3.7	2.2	28.15	28.6	Stingl et al., 2013
Ukraine	102	-	-	_	25	Weinshilboum, 2003
Turkish	344	-	6.4	25.3	2	Giardina et al., 2018
**Africa**		-		-	-	
West Africa	153	4	42	-	50	Evans and Relling, 2004
Congo	418	-	-	-	55^¥^	Lynch and Price, 2007
Ghana	800	-	-	-	48	Zanger and Schwab, 2013
Botswana	570	-	-	22		Nebert et al., 2013
Mozambique	360	5.7	41	-	42.6^¥^	Lonsdale et al., 2013
Nigeria (Hausa)	100	-	-	42	-	Thorn et al., 2010
Nigeria (Igbo)	100	-	-	38	-	Thorn et al., 2010
Nigeria (Yoruba)	100	-	-	42	-	Thorn et al., 2010
Tanzania	256	-	-	-	36^¥^	Desta et al., 2021
Ethiopia	285	-	-	-	31.4^¥^	Rendic, 2002
Kenya (Kikuyu)	102	-	-	34	-	Thorn et al., 2010
Kenya (Luo)	100	-	-	37	-	Thorn et al., 2010
Kenya (Maasai)	152	-	-	35	-	Thorn et al., 2010
Zimbabwe (San)	64	-	-	40	-	Thorn et al., 2010
Zimbabwe (Shona)	100	-	-	38	-	Thorn et al., 2010
Cameroon	75	-	-	-	37^¥^	Hanna et al., 2000
South Africa	163	-	-	-	36^¥^	Hanna et al., 2000
South Africa (Venda)	81	-	-	36		Thorn et al., 2010
South Africa (Xhosa)	109	-	-	-	20^¥^	Zanger et al., 2008
South Africa (MA)	67	-	-	-	23^¥^	Zanger et al., 2008
Uganda Bantus	58	-	-	25.9	-	Sarfo et al., 2014
**Asia**		-	-	-		
Thai	100	6	3.6	-	32^¥^	Kharasch and Greenblatt, 2019
Chinese	567	2.8	3.2	-	25.9^¥^	Chaivichacharn et al., 2020
Japanese	530	4.7	9.3	16.4	-	Bank et al., 2019
Han Chinese	386		9.1	18.4	1.8	Giardina et al., 2018
Malaysian Malay	196	0.8	7.6	25.4	4.6	Kim et al., 2017
Malaysian Chinese	165	1.3	6.4	13.9	10.2	Kim et al., 2017
Malaysian Indian	63	4.1	9.9	18.5	5.9	Kim et al., 2017
South Indians	135	-	-	-	44	Bielinski et al., 2014
Koreans	374	-	-	16.4	-	Dolgin, 2011
Indonesia (Timorian)	109	-	56.8	41.7	46.9	Drozda et al., 2018
Taiwanese	68	-	11.8	16.2	0	Hesse et al., 2004
Mongolian	200	-	9	21	-	Ekins et al., 1998; Giardina et al., 2018
Papa New Guinea	172	0	0	65	0	Lang et al., 2001
**America**		-	-	-	-	
Columbian Mestizo	250	4.4	15.2	18	14.4	Wang et al., 2003a
Central American Mestizo	362	-	7.3	23.1	2.3	Wang et al., 2003a
Chilean Mestizo	438	-	-	35	-	Wang et al., 2003a
Hispanic–American	77	3	35	-	37	Evans and Relling, 2004
African American	85	2	37	-	36	Evans and Relling, 2004

¥ In the manuscript, it is referred to as CYP2B6*6 but according to the Pharmacogene Variation Consortium (Pharmvar) website it should be CYP2B6*9, CYP2B6*6 is a combination of CYP2B6*4 & CYP2B6*9. (-) Allele not verified.

## Population Disparity in the Use of CYP2B6 Substrates and Consequent Exposure to Substrate-Specific Adverse Drug Reaction (ADR)

### Efavirenz and Nevirapine

Efavirenz (EFV) is classified as an effective non-nucleoside reverse transcriptase inhibitor used in the treatment of HIV infection^1^^,^^2^. As part of the highly active antiretroviral therapy, EFV is used in combination with other nucleoside reverse transcriptase inhibitors (NRTIs)^1, 2^. EFV, which is presented in the form of 600 mg once daily or in a reduced dose of 400 mg oral tablets, is metabolized mainly by hepatic CYP2B6. EFV therapy is limited due to its narrow therapeutic window, thus, there exist a small difference between therapeutic and toxic doses. ADRs which are linked to the use of EFV includes increased risk of neurotoxicity, neuropsychiatric disorders, sleep disorders, high cholesterol level and drug induced liver disease (Cohen et al., 2009; Yimer et al., 2011; Aminkeng et al., 2014; Sarfo et al., 2014; Dhoro et al., 2015).

CYP2B6 genotype is a strong predictor of high systemic exposure to EFV in HIV infected patients. Table 3 presents studies on associations between CYP2B6 genotype and the occurrence of CYP2B6 substrate related ADRs in various ethnicities. According to reports across ethnicities, patients harboring the CYP2B6^*^6 (516G>T, 785A>G*)* and the CYP2B6^*^18 (983T>C) variants experience reduced metabolism of EFV and increased exposure to the drug (Table 3). Patients with the homozygote 516TT and/or 785GG genotype as well as those with the 983CC genotype (poor metabolizers) experience significant increase in EFV plasma concentration and reduced clearance (Table 3). High exposure to EFV increases the risk of ADRs in these patients (Gounden et al., 2010; Mukonzo et al., 2013; Sarfo et al., 2014). Amongst all CYP2B6 substrates, EFV is the most studied drug with diverse forms of ADRs reported from various ethnicities. EFV is among top drugs causing ADRs in HIV patients in Africa, some part of Eastern Europe, and Asia (Manosuthi et al., 2013; Birbal et al., 2016; Rajman et al., 2017).

**Table 3:** CYP2B6 polymorphisms and adverse drug reactions reported amongst patients in various ethnicities.

**Substrate**	**Subjects**	***N***	**CYP2B6 genotype**	**Predicted functional effect on CYP2B6 enzyme activity**	**Patient exposure to the drug**	**Frequency of allelic variants (%)**	**Population**	**Adverse drug reaction**	**References**
Efavirenz	HIV/TB	185	CYP2B6*6/*6	↓ Activity	Higher exposure	45	Zimbabwe	Central nervous system adverse events(CNS) including insomnia, severe headaches, vivid nightmares, drowsiness, ataxia, dystonia and dizziness.	Patel and Patel, 2018
	HIV, TB-HIV co-infected patient	353	CYP2B6 516TT	↓ Activity	Higher exposure	31.6	Ethiopians	Anti-retroviral and anti-tuberculosis drug induced liver injury in TB-HIV co-infected patients.	Agency, 2017; Giardina et al., 2018
	HIV	285	CYP2B6 516TT	↓ Activity	Higher exposure	31	Ethiopians	Higher risk of drug induced liver injury!!break (DILI)	Agency, 2017; Schurig et al., 2018
	HIV	800	CYP2B6 516TT	↓ Activity	Higher exposure	48	Ghanaian	Neuropsychiatric toxicity	Stingl et al., 2013
	HIV	134	CYP2B6*6/*6	↓ Activity	Higher exposure	8.2	Thai	Increase risk of hepatotoxicity	Weinshilboum, 2003; Evans and Relling, 2004; Lynch and Price, 2007
	HIV/AIDS	1,147	CYP2B6 G516TT	↓ Activity	Higher exposure	38, 21.9	Mixed population European American, African American, Hispanics	Central nervous system toxicity	Zanger and Schwab, 2013
	HIV/AIDS	373	CYP2B6 516TT	↓ Activity	Higher exposure	30, 37	Mixed population (Black & White)	Central nervous system related effects!!break and 131 patients withdrew from therapy within the first 3 months	Nebert et al., 2013
	HIV	197	CYP2B6 516TT	↓ Activity	Higher exposure	30	Ugandans	Neuropsychiatric symptoms. High incidence of vivid dream, sleepwalking, insomnia and tactile hallucination	Thorn et al., 2010; Lonsdale et al., 2013
	HIV/TB patients	473	CYP2B6 516GT!!break CYP2B6 516TT	↓ Activity	Higher exposure	35.5	Tanzanians	Development of efavirenz based HAART liver injury	Agency, 2017; Desta et al., 2021
	HIV adults	142	CYP2B6 516GT!!break CYP2B6 516TT	↓ Activity	Higher exposure	32	South Africans	High efavirenz level associated with severed sleep disturbance	Rendic, 2002
	HIV	80	CYP2B6 516GT!!break CYP2B 516TT	↓ Activity	Higher exposure	43	South!!break Africans	Higher EFV concentration and early neuropsychiatric side effects (presence of hallucinations or psychotic episodes)	Hanna et al., 2000
	HIV	191	CYP2B6 516GT, CYP2B6 516TT	↓ Activity	Higher exposure	49	Mixed population (Caucasian 162, African 23, Asiantic four, others two)	CNS related symptoms such as disturbances in consciousness, mood disorders, headaches, sleep disturbances, cognitive and attention disturbances, eating disturbances and dizziness	Zanger et al., 2008
	HIV/AIDS	1330	CYP2B 516GT/TT	↓ Activity	Higher exposure	77, 25	Mixed population African ancestry (372), European ancestry (958)	Increase in cholesterol levels, Increased risk of neurotoxicity, CNS depression and neuropsychiatric disorders, Increased risk of fatigue and sleep disorder and Increased risk of hepatotoxicity and drug-induced liver injury	Sarfo et al., 2014
	HIV	32/90	CYP2B6 516TT	↓ Activity	Higher exposure	32	Mixed population	Increased likelihood of central nervous system disease	Kharasch and Greenblatt, 2019
	HIV	235	CYP2B6 516TT	↓ Activity	Higher exposure	26	Swiss	Neuropsychological toxicity	Chaivichacharn et al., 2020
	HIV patients	105	CYP2B6*1/*18, CYP2B6*18/*18	↓ Activity	Higher exposure	5.1	Mozambicans	Associated with severe cutaneous!!break adverse event such as Steven-Johnson syndrome and toxic epidermal necrolysis	Bank et al., 2019
Nevirapine	HIV	672	CYP2B6*18	↓ Activity	Higher exposure	18	Malawians!!break Ugandans	Hypersensitivity such as nevirapine induced-Stevens–Johnson syndrome (SJS)	Kim et al., 2017
	HIV	105	CYP2B6 516TT!!break CYP2B6 983CC	↓ Activity	Higher exposure	55.6!!break 18.5	Mozambicans	Patients with Nevirapine-induced SJS/toxic epidermal necrolysis (TEN)	Bank et al., 2019
Cyclophosphamide	non-Hodgkin's lymphoma	567	CYP2B6 516TT!!break CYP2B6 785AG (*4)	↓ Activity	Reduce exposure to 4-hydroxycy-cyclophosphamide	25.9!!break 32	Chinese	Lower risk of grade 2–4 toxicities and!!break poor treatment outcome	Bielinski et al., 2014
	Breast Cancer	230	CYP2B6*2 CYP2B6*4!!break CYP2B6*5!!break CYP2B6*9	↑ Activity	High exposure	227!!break 15!!break 30	Mixed population (European 97, South Asian 2, East Asian <1)	Leucopenia and neutropenia associated with dose delay	Hesse et al., 2004; Dolgin, 2011; Drozda et al., 2018
	Breast cancer	Case report	CYP2B6*7	↑ Activity	Higher exposure to 4-hydroxycy-cyclophosphamide	18.4	Han Chinese	Severe and prolonged hepatotoxicity	Ekins et al., 1998; Dolgin, 2011; Drozda et al., 2018
	Chronic lymphocytic leukemia (CLL)	428	CYP2B6*1/*6!!break CYP2B6*6/*6	↓ Activity	Reduced exposure	22	UK clinical trial	Decrease risk of drug toxicity. Toxicity included neutropenia, thrombocytopenia, anemia, mucositis, and alopecia	Lang et al., 2001
	Leukemia patients on stem cell transplant	107	CYP2B6*4!!break CYP2B6*2!!break CYP2B6*6 (donor GG genotype)	↑ activity	Higher exposure	53!!break 16, 50	Mixed population	Oral mucositis, (*4), hemorrhagic cystitis (*2),Veno-occlusive disease of the!!break liver (*6),	Wang et al., 2003a
Methadone	Breast cancer	166	CYP2B6 516GT,!!break CYP2B6516TT	↓ Activity	High exposure	27	Brazilian	High risk of severe levels of asthenia and arthralgia	Ekins et al., 1998; Wang et al., 2003b
	Fatalities cases due to methadone	380	CYP2B6*9 CYP2B6*5	↓ Activity	High exposure	27	Caucasians	Methadone fatalities	Lee et al., 2003; Wang et al., 2003b; Liptrott et al., 2009
	Opioid dependent males	148	CYP2B6*6	↓ Activity	High exposure	25.4	Malays	Lower pain threshold, increase severity of pain	Lee et al., 2003; Liptrott et al., 2009

*↓, Decrease; ↑, Increase*.

Nevirapine (NVP) based antiretroviral therapy is also used as a first line regimen for HIV infection. Its usage is limited due to side effects including hepatotoxicity, fever, Steven-Johnson syndrome and toxic epidermal necrolysis (Ciccacci et al., 2013; Aminkeng et al., 2014). In addition to other factors such as weight and gender, *CYP2B6* genotype influences patient response to NVP (Srivastava et al., 2010; Rajman et al., 2017; Yoon et al., 2020). These variants, CYP2B6 516G>T and 983T>C (CYP2B6^*^18) also impact patient exposure to NVP as reported by few studies from different ethnicities (Uttayamakul et al., 2010; Calcagno et al., 2012; Vardhanabhuti et al., 2013; Mhandire et al., 2015; Giacomelli et al., 2018). A reduced metabolic activity of the CYP2B6 enzyme leading to increased exposure of the patient and subsequent ADRs are observed (Yoon et al., 2020) (Table 3). NVP induced hypersensitivity reactions in HIV patients (Ciccacci et al., 2010; Carr et al., 2014). The variant allele of CYP2B6^*^18 and CYP2B6^*^4 were associated with the occurrence of Stevens-Johnson syndrome (SJS) among HIV patients (Ciccacci et al., 2013; Carr et al., 2014). Studies suggest dose reduction in patients harboring CYP2B6 poor metabolizer variants (Schipani et al., 2011; Morales-Pérez, 2021).

Interestingly, the use of EFV and NVP for HIV treatment is variable among populations. Highly active antiretroviral therapy (HAART) that includes EFV or NVP is mostly used in low and middle income countries (LMICs) including countries in Africa, Asia and some part of Eastern Europe (Gokengin et al., 2018; Ndashimye and Arts, 2019). Some studies have examined the reasons for such disparity in the use of EFV. It was observed that countries with higher income had greater access to novel drugs and new drug classes as first-line treatment than countries with low income (Gokengin et al., 2018; Ndashimye and Arts, 2019). Although most HAART regimens are given free to patients in all countries, the high level of donor dependency in LMICs might be a major obstacle to accessibility to new, expensive, more quality and less toxic drugs (Gokengin et al., 2018). The high cost of new first-line drugs with better tolerability and lower toxicity influences the choice of treatment regimen, with high income countries (HICs) choosing regimens that include new drugs compared to LMICs who depend on cheaper regimens (Gokengin et al., 2018). We observe an association between countries depending on cheaper drugs (EFV) and a high frequency of CYP2B6^*^6 which enhances the risk of EFV related ADRs in these populations (Africans, Papua New Guineans and African Americans with low income/incomplete insurance coverage). Intraethnic variability of CYP2B6 alleles within the African population, further indicates differences in the risk profiles of occurrence of CYP2B6^*^6 and CYP2B6^*^9-EFV-related ADRs between these African countries. This genomic diversity of African populations has been observed for many genes (Rajman et al., 2017) and highlights the potential error in generalizing the likely response to medicines of populations on the African continent (Ampadu et al., 2016). HIV infected individuals in LMICs are more likely to suffer from EFV-related ADRs than those in HICs.

Even though many LMICs have transited from the use of EFV and NVP to the use of the HIV-1 integrase inhibitor dolutegravir (DTG) which is assumed to be less expensive and less toxic (Vitoria et al., 2018), DTG has its own limitations as confirmed by the U.S. Food and Drug Administration (FDA). DGT may be unsuitable for patients with hepatitis B and C infection due to elevated levels of hepatic enzyme (Vitoria et al., 2018). Risk of hypersensitivity reactions and development of renal failure may be observed (Vitoria et al., 2018). New-onset or worsening hepatic or renal toxicity with longer cumulative exposure is a potential risk (Vitoria et al., 2018). In pregnancy, there are concerns that the baby may be harmed, thus women are recommended to take effective birth control while on the drug (Vitoria et al., 2018; Zash et al., 2019; Alhassan et al., 2020). Weight gain was observed in patients treated with DTG compared to EFV in clinical trials performed in sub-Saharan Africa and studies in Europe and North America. Therefore, long term use of DTG is still under concern (Kouanfack et al., 2019; Bourgi et al., 2020; Sax et al., 2020). A recent communication in December 2020 by Siedner et al. found that patients with drug resistant mutation in the reverse transcriptase are unlikely to benefit from the HIV integrase inhibitor DTG (Siedner et al., 2020). That implies EFV and NVP might still be in use for specific group of patients (Vitoria et al., 2018).

The occurrence of other diseases, including hepatitis and liver disease, are more common among HIV patients in LMICs (Labarga et al., 2007; Su et al., 2018). Unfortunately, much care is not given to HIV patients who have comorbid conditions, thus increasing the risk of ADRs amongst these patients (Labarga et al., 2007; Wu et al., 2017; Su et al., 2018). For example, patients with liver disease and hepatitis may experience enhanced hepatotoxicity due to EFV (Agbaji et al., 2013; Wu et al., 2017; Nampala et al., 2018; Su et al., 2018). Certain preexisting conditions, such as mild psychiatric disorder, may be exacerbated by EFV. Patients in LMICs may be vulnerable to ADRs not just because of the choice of regimen or their CYP2B6 genotype, but also because of comorbidity conditions. As such individualized prescriptions taking into account the pharmacogenetic profile, patient vulnerability caused by comorbid disease, and the toxicity profile of the drug will help to reduce ADR amongst these group of individuals in any part of the world.

### Cyclophosphamide

Cyclophosphamide (CP) is an anticancer agent that is widely used in the treatment of pediatric and adult malignancies. CP is also known as an antirheumatic and anti-inflammatory drug (Brummaier et al., 2013; Sevko et al., 2013). CP is a prodrug that requires enzymatic bioactivation to produce its therapeutic function (Raccor et al., 2012). The formation of 4-hydroxycyclophosphamide (4-OHCP) is catalyzed by CYP2B6, CYP2C9, CYP2C19, CYP3A4 and CYP3A5. CYP2B6 and CYP2C19 confer the highest metabolic activity for the bioactivation of CP (Roy et al., 1999; Griskevicius et al., 2003; Raccor et al., 2012).

There is a significant variation in the plasma levels of CP ranging from 1.0 to 12.6 L/h (Batey et al., 2002; de Jonge et al., 2005). According to *in vitro* studies, it is clear that genetic polymorphisms in *CYP2B6* gene and interindividual differences in the CYP2B6 enzymatic activity are associated with variability in CP efficacy (Roy et al., 1999; Xie et al., 2003; Hofmann et al., 2008). Very limited clinical studies have evaluated the impact of CYP2B6 variants on patient exposure to CP, instead, many studies have investigated the influence of patient genotype on therapeutic outcome or treatment efficacy of CP. However, Shu et al., showed a significant association between CYP2B6 genotype and exposure to 4-OHCP amongst 567 patients with non-Hodgkin lymphoma (Table 3). Reduced expression and catalytic activity of CYP2B6 protein was found among homozygous and heterozygous carriers of CYP2B6^*^6, ^*^29, and ^*^30 (Table 3). Patients with the 785G allele (^*^6) showed the worst treatment response (Shu et al., 2017). In breast cancer patients, a higher incidence of dose delay due to toxicity was observed among carriers of the CPY2B6 ^*^2 and ^*^5 (Bray et al., 2010). Furthermore, CYP2B6^*^2, ^*^4, ^*^8, and ^*^9 alleles where associated with worse treatment outcome (Bray et al., 2010). In another study, an association was observed between CYP2B6^*^6 allele and inferior treatment response in patients with chronic lymphocytic leukemia. Patients harboring at least one CYP2B6^*^6 allele were less likely to achieve complete response to CP and fludarabine combination therapy due to impaired cytoreduction (Johnson et al., 2013).

Decreased risk of drug toxicity (neutropenia, thrombocytopenia, anemia, mucositis and alopecia) was observed in carriers of CYP2B6 ^*^1/^*^6 and CYP2B6^*^6/^*^6 genotypes (Table 3). Considering the fact that CYP2B6^*^6 is a decreased function allele, these patients seem to experience less toxicity and impaired cytoreduction due to the inability of the variant enzyme with reduced activity to convert CP to the active anticancer metabolite which is also responsible for toxicity. A significant association was observed between CYP2B6 785A>G (^*^4) and shorter progression free survival (Falk et al., 2012) in patients with multiple myeloma. Patients with the 785GG genotype had the worse outcome compared to patients with the wild type allele. In another study involving lymphoma patients who were placed on high dose CP prior to hematopoietic stem cell transplant, patients with the CYP2B6^*^1/^*^5 genotype had a higher 2 years relapse and decrease overall survival than patients with the wild type genotype (Bachanova et al., 2015). Though the CYP2B6^*^5 variant is designated as normal function, *in vitro* studies of CP revealed reduced conversion of CP in human liver microsomes and recombinant expressed CYP2B6^*^5 variants confer 50% decrease in the formation of 4-OHCP compared to the wild type enzyme (Helsby et al., 2010; Raccor et al., 2012). Most recently, CP related toxicity was associated with *CYP2B6* variants in patients with leukemia after HLA-identical hematopoietic stem cell transplantation (Rocha et al., 2009). Donor CYP2B6^*^6 was associated with Veno-occlusive disease of the liver meanwhile recipient CYP2B6^*^2 and ^*^4 were associated with hemorrhagic cystitis and oral mucositis correspondingly (Rocha et al., 2009). High risk of severe levels of asthenia and arthralgia was observed amongst breast cancer women with CYP2B6 516GT and 516TT genotype placed on FAC-D combination therapy (Paula et al., 2020). Some studies also evaluated the role of CYP2C19 variants alongside with CYP2B6 in the efficacy and toxicity of cyclophosphamide. Even though few studies could not find any association between CYP2B6 or CYP2C19 and treatment outcome and/or toxicity of CP, differences in the study design, ethnicity, physiological or disease condition as well as power of the studies might have a role in the experimental outcome. However, it appears that these two pharmacogenes (*CYP2B6* & *CYP2C19*) play a significant role in the bioactivation and efficacy of CP.

Variability in *CYP2B6* polymorphisms between populations indicates that cancer patients in various ethnicities will respond variably to CP therapy. For example, CYP2B6^*^9 and CYP2B6^*^2 variants which are associated with toxicity and worse treatment outcome were not detected among 172 individuals from Papua New Guinea (Mehlotra et al., 2006; Bray et al., 2010), thus, cancer patients in this region are less likely to experience CYP2B6 pharmacogenetic associated-CP toxicity. Meanwhile, in Mozambique and Germany, patients are more likely to experience toxicity and worse treatment outcome due to the frequency of CYP2B6^*^9 (42.6 and 28.6%) in these populations (Tables 2, 3). Furthermore, reduced CP toxicity and impaired cytoreduction are likely to occur in populations with high frequency of CYP2B6^*^6 (Tables 2, 3).

According to research, the number of cancer survivals vary between and within countries. These differences are partly due to limited access to high quality and effective treatment in some part of the world (Newton et al., 2010; Eniu et al., 2019). CP is included in the WHO list of essential medicine (Bazargani et al., 2015). However, the majority of cancer patients living in LMICs have limited access to essential medicine and sometimes imported medication are either counterfeits or of poor quality (Fernandez et al., 2011; Ruff et al., 2016). Cancer care is not included in most resource limited settings due to high cost, as such, patients have to pay for cancer treatment out of their pocket (Eniu et al., 2019). In Asia, the number of death in a year ranged from 12% in Malaysia to 45% in Myanmar amongst cancer patients and those with low income were at risk of adverse outcomes even at early disease stages (ACTION Study Group, 2017). Important barriers to availability and affordability of anticancer drugs include lack of governmental reimbursement, allocation of healthcare budgets, generic and biosimilar products as well as the right to patent (Renner et al., 2013; Eniu et al., 2019). Therefore, patients in these regions are likely to experience drug resistance, treatment failure and more ADRs than those in HICs (Newton et al., 2010).

Additionally, increase number of adverse outcome is expected to occur in Africa, Asia and some parts of Europe, where other diseases such as HIV, tuberculosis and hepatitis are found amongst cancer patients who are self-sponsored without any health care coverage and reduced compliance to medication due to lack of funds and timely treatment (ACTION Study Group, 2017; Eniu et al., 2019). Therefore, in addition to other confounders, increased accessibility to essential medicine, availability of health care coverage, special care for patients with comorbidity and genotyping of patients may improve therapy.

### Bupropion

Bupropion, generally used as an antidepressant, was later recommended as a non-nicotine treatment for smoking cessation. It is approved for treatment of nicotine dependence in patients with tobacco use disorder (Jorenby et al., 1999; Schnoll and Lerman, 2006). It acts by increasing dopaminergic and noradrenergic transmission via the blockage of neurotransmitter reuptake at the synapse thereby antagonizing the effects of nicotine acetylcholine receptor leading to nicotine withdrawal (Paterson et al., 2007). Also, bupropion is prescribed alone or in combination with other antidepressants for treatment of major depressive disorder and seasonal affective disorder. It is used as an adjunctive medication to reverse antidepressant-associated sexual dysfunction and to improve the efficacy of other antidepressants in partial or non-responders (Fava et al., 2005). Both bupropion and the active metabolite S-hydroxybupropion are responsible for the antidepressant and smoking cessation effect of bupropion (Zhu et al., 2012; Laib et al., 2014).

*In vitro* studies revealed variability in the rate of metabolism of S-bupropion by various CYP2B6 variants (Wang et al., 2020). Studies from different ethnicities show that the CYP2B6 allelic variants ^*^4, ^*^6, and ^*^18 are associated with altered bupropion metabolism (Kirchheiner et al., 2003; Zhu et al., 2012; Benowitz et al., 2013). Depending on the patient's genotype, they can experience either increased or decreased exposure to the active metabolite hydroyxbupropion compared to wild type (Kirchheiner et al., 2003; Benowitz et al., 2013). Reportedly, patients harboring at least one reduced function allele had reduced exposure to the active metabolite and a lower clearance of the parent drug (Kirchheiner et al., 2003). Meanwhile, those with at least one increased function allele have increased exposure to hydroxyl active metabolites and a higher clearance of the parent drug (Kirchheiner et al., 2003).

Some studies have linked patient genotype to the efficacy of bupropion as a smoking cessation agent. In a smoking cessation trial involving 326 Europeans, a significant rate of abstinence was observed among patients with the CYP2B6^*^1/^*^6 and CYP2B6^*^6/^*^6 genotype (Lee et al., 2007). The CYP2B6^*^4 variant allele was associated with lower success rate in bupropion therapy. Specifically, patients with the AA genotype (wild type) succeeded in ceasing smoking compared to those with the variant genotype 785AG and 785GG (Tomaz et al., 2015). The increase function alleles might have led to higher clearance of the drug as well as reduced efficacy. In another clinical trial, bupropion drug gene interaction indicated that individuals with at least one T allele of CYP2B6^*^5 (1459CT, TT) and DRD2-Taq1 A2/A2 genotype had higher odds of abstinence (David et al., 2007). There are some differences in experimental outcome, for example, in a clinical trial involving 540 African American light smokers, a direct association between CYP2B6 genotype and smoking cessation was not observed, according to the authors, poor adherence amongst the participants might have led to the observed results. Most studies did not assess the impact of CYP2B6 polymorphisms on the antidepressant effect of bupropion. Instead, many focused on the CYP2B6 pharmacogenetics of bupropion in smoking cessation (Pharmgkb, 2000; Clark et al., 2012).

Compared to HICs, mental health in LMICs is still limited with low number of clinical trials and extremely low number of patients receiving treatment (Sankoh et al., 2018; Chibanda et al., 2020). Individuals with serious mental health problems in Nigeria cannot have access to health care due to the limited number of resources and mental health staff (Chibanda et al., 2020). Approximately, 300 psychiatric doctors serve a population of about 200 million people. Currently, these issues are being addressed by the Nigerian government via the forthcoming Mental Health and substance abuse bill (Brathwaite et al., 2020; Ugochukwu et al., 2020). Another study involving 683 prescriptions and case records of patients who were placed on antidepressants showed that tricyclic antidepressants were the most prescribed drugs (61.3%), followed by selective serotonin re-uptake inhibitors (38.7%) in Nigeria (Oyinlade, 2017). Amitriptyline hydrochloride, fluoxetine, clomipramine, escitalopram, and imipramine hydrochloride are the antidepressants found on the WHO list of essential medicine for Nigeria (WHO, 2017)^3^. In Zimbabwe, a research made in 2017 indicated that psychiatrists sometimes experience shortage of drugs which results in relapse among patients (Kidia et al., 2017). The antidepressants available were old generation drugs with many side effects (Kidia et al., 2017). According to one of the psychiatrists, more attention is being given to people with HIV than those with mental health, furthermore mental health policies were never implemented because of inadequate funding (Kidia et al., 2017). The African Mental Health Research Initiative group aims to create awareness and strengthen the mental health sector via research and capacity building in Sub-Saharan Africa (Chibanda et al., 2020; Langhaug et al., 2020).

The Global Health Observatory (GHO) data indicates the availability of bupropion in various countries (GHO/WHO, 2007). In some countries bupropion is available in the pharmacy with prescription. In others, it is found in general stores without prescription (Langhaug et al., 2020). Meanwhile, for some countries, data for the use of bupropion is not available. For example, GHO data shows that in Germany, France, Australia, Canada, United States of America, Congo, and India bupropion is available in pharmacies with prescription, whereas in Nigeria, it is found in general stores without prescription, in Malaysia, Ukraine, Zimbabwe, and Cameroon, GHO 2012 data shows that bupropion was present in pharmacy with prescription. In Hungary, Algeria, Tanzania, Mali, no data was available from 2007 to 2018 (GHO/WHO, 2007). Compared to HICs, the shortage of medication faced in LMIC is likely to increase relapse and the use of old drugs with more side effects can aggravate ADRs among patients.

Though bupropion is cost effective and widely used in most countries, its application as a smoking cessation agent is still very limited in some LMICs. The MPOWER tobacco control measures put in place by the World Health Organization (WHO) in 2005 have greatly helped many countries to discourage the use of tobacco and to help users quit tobacco addiction^4^. MPOWER measure is a tobacco free initiative established by WHO for defeating the global tobacco epidemic via **M**onitoring tobacco use, **P**rotecting people from tobacco use, **O**ffering help to quit, **W**arning about dangers of tobacco, **E**nforcing bans on advertising and sponsorship and **R**aising taxes on tobacco in all parts of the world (Batini et al., 2019). According to a recent report by Batini et al., the application of the MPOWER measures vary across continents and countries with a marked discrepancy observed between LMICs, and HICs, where cost, governmental buy in and resources influence implementation of the various measures (Batini et al., 2019). Using the African continent as an example, authors report that pharmacotherapy for smoking cessation is scarce in Nigeria with few pharmacies offering nicotine replacement therapy. In Zimbabwe, there are currently no smoking cessation services in the public sector except for private sectors, which is offered at very high cost (NRT 130USD/week, varenicline 250USD/month). In Tanzania, nicotine replacement therapy is available and smoking cessation therapy has been introduced into the methadone clinic in Dar es Salaam (Batini et al., 2019). However, the use of non-nicotine therapy such as bupropion is still lacking in these countries.

Depression seems to coexist with many diseases that are common in both high and LMICs. In Sub Saharan Africa, depression is common among HIV patients and people with disability (Mayston et al., 2020). In India, high prevalence of depressive episodes were found among diabetic patients (Kanwar et al., 2019). In a comparison between Ethiopian and German cancer patients, depression and anxiety were found in both groups, but a little higher among uneducated Ethiopian patients (Wondie et al., 2020). Considering the fact that some HIV and cancer patients in LMICs are not able to afford treatment, depression might be high among these group of patients. It is well-established that CYP2B6 genotype is a significant contributor to variability in hydroxybupropion and bupropion levels, influencing the efficacy of bupropion therapy in smoking cessation patients. Knowledge on CYP2B6 genotype in consideration with other factors might give a clue on patients who are likely to benefit from therapy. Constant supply of bupropion could help to reduce relapse among patients in most LMICs. Treatments of other comorbidities that are risk factors of depression have the potential to improve therapy and reduce the number of depressive patients worldwide. There is need to create awareness in the area of mental health via research and innovation, including capacity building, training more mental health staff and psychiatric doctors in LMICs.

### Ketamine

Ketamine is a World Health Organization Essential Medicine widely used for perioperative, acute and chronic pain, and sedation. It is used either solely or in combination with opioids for the management of acute post-operative and chronic refractory pain (Laskowski et al., 2011). Its use in the management of status epilepticus, bipolar disorder, suicidal behavior, major depressive disorder and treatment resistant depression has been demonstrated (Yeh et al., 2011; Synowiec et al., 2013; Pizzi et al., 2017; Borsato et al., 2020). Ketamine acts as a non-competitive antagonist of the N-Methyl-D-aspartic acid receptor blocking its action, thereby preventing the development and chronification of pain (Noppers et al., 2010). CYP2B6 is among other enzymes (CYP2C9, CYP3A4) involved in the hepatic N-demethylation of ketamine to norketamine (Yanagihara et al., 2001; Hijazi and Boulieu, 2002; Portmann et al., 2010; Desta et al., 2012; Palacharla et al., 2018). Although ketamine has a large therapeutic window, its use is limited due to low efficacy and huge interindividaul variability in treatment response including ADRs that require cessation of therapy (Kvarnström et al., 2004; Noppers et al., 2010; Laskowski et al., 2011; Hardy et al., 2012; Perez-Ruixo et al., 2020). Ketamine has been associated with increased blood pressure, alteration of speech, muscular discoordination, euphoria, hallucination, loss of consciousness, seizure, nausea, out of body experience, hypothermia, traffic accident or drowning and irrational behavior (Iyalomhe and Iyalomhe, 2014; Lonnée et al., 2018; Gajewski et al., 2020).

Variability in the expression and catalytic activity of CYP2B6 variant enzymes results in differences in the hepatic clearance of ketamine (Wang et al., 2018). Individual differences in hepatic blood flow leading to differences in patient clearance of the drug has also been implicated (Yanagihara et al., 2001). The presence of the CYP2B6^*^6 allele led to a decrease in the intrinsic clearance of ketamine of up to 89% in human liver microsomes and 55% in cDNA-expressed CYP2B6 protein *in vitro* (Yanagihara et al., 2001). The CYP2B6^*^1/^*^1 (wildtype) genotype confers a 6-fold higher clearance of both enantiomers of ketamine compared to the CYP2B6^*^6/^*^6 genotype (Li et al., 2013). In a study involving patients with chronic opioid-refractory pain, the plasma clearance of ketamine was lower in patients with CYP2B6^*^6/^*^6 and CYP2B6^*^1/^*^6 genotype compared to patients with CYP2B6^*^1/^*^1 genotype (Li et al., 2015). Although there were no direct associations between the genotype of the patients and the occurrence of ADRs, drowsiness and hallucination were more often observed in patients with lower clearance than those with higher clearance. The authors hypostatized that higher plasma concentrations due to reduced clearance may have predisposed patients to ketamine ADRs. *In vitro* studies further demonstrate that CYP2B6 variants confer variability in the metabolism of ketamine. S-ketamine metabolism ranged from CYP2B6^*^1 (wildtype) > CYP2B6^*^4 > CYP2B6^*^26, CYP2B6^*^19, CYP2B6^*^17, CYP2B6^*^6 > CYP2B6^*^5, CYP2B6^*^7 > CYP2B6^*^9, respectively (Wang et al., 2018). This indicates that patients harboring different CYP2B6 variants will respond differently to the drug (Borsato et al., 2020). Genetic variations in *CYP2B6* and other CYPs including *CYP3A4* might result in different plasma concentration of ketamine and its metabolites. Therefore, knowing the genotype of the patient prior to prescription could help to address individual needs and reduce ADRs.

There is a huge difference in availability and application of anesthesia between high and LMICs as reviewed by Dolman et al. Factors influencing these differences include limited physician anesthesiologists and nurse anesthetists, insufficient anesthesia equipment and infrastructure, patient comorbidities and late presentations (LeBrun et al., 2014; Dohlman, 2017). In LMICs, ketamine is the only anesthetic drug in many hospitals as compared to HICs, where it is used as an adjunct in combination with other anesthetics (Dohlman, 2017). LMICs are lacking in the provision and training for safe anesthesia practice. In a cross-sectional survey made in Zimbabwe involving 42 hospitals, the number of specialist physician anesthetics were limited (Lonnée et al., 2018). Further, 19% of the nurse anesthetists have had no formal training (Lonnée et al., 2018). In Nigeria, intravenous ketamine is used as general anesthesia in parts of the country where anesthesiologist's services are scarce. However, few patients experience adverse effects including high blood pressure, priapism, emergent delirium, tachycardia, disorientation and confusion (Iyalomhe and Iyalomhe, 2014). In a recent survey conducted in Tanzania, Malawi and Zambia, ketamine was widely used in many of the hospitals to compensate for shortages of other forms of anesthesia. Anesthesia care in these countries were performed by non-physician anesthetists, some of whom had no formal training. Shortage of staff, interrupted access to electricity and water for some facilities and lack of functional anesthesia machines were reported (Gajewski et al., 2020). Though ketamine is considered safe, its application by untrained personal might lead to abnormal doses and contribute to adverse effects in these populations.

### Methadone

Methadone is a synthetic opioid utilized for the treatment of chronic, acute and neuropathic pain. It is also used as an analgesic to treat pain in cancer patients and as a maintenance therapy for opiate addiction (Chou et al., 2009; Parsons et al., 2010; Kharasch, 2011). In opioid use disorder, methadone reduces the painful symptoms of opiate withdrawal and relieves drug craving by acting on the opioid receptor in the brain. Methadone is administered as a racemate consisting of the *S*-and *R*-enantiomers (Crettol et al., 2005; Ansermot et al., 2010). Methadone is metabolized in the liver via N-demethylation performed by various cytochromes including CYP3A4 and CYP2B6. CYP2B6 confers enantioselectivity for the S-enantiomer (Yang et al., 2016), further, the influence of CYP2B6 variants on the S-enantiomer has been reported (Kharasch and Crafford, 2019).

*In vitro* studies demonstrate differential metabolism and clearance of methadone by CYP2B6 variants ranging from CYP2B6^*^4 ≥ CYP2B6^*^1 > CYP2B6^*^5 > CYP2B6^*^9 ≥ CYP2B6^*^6 (Gadel et al., 2015). CYP2B6 genotype influences the plasma levels of both enantiomers (Victorri-Vigneau et al., 2019). Also, decreased clearance and high plasma concentration of methadone enantiomers was observed in patients with CYP2B6^*^1/^*^6 and CYP2B6^*^6/^*^6 compared to controls (Eap et al., 2007; Kharasch et al., 2015; Kringen et al., 2017; Talal et al., 2020). In a study involving 125 methadone fatality cases, the frequency of CYP2B6^*^9 was high in the methadone group compared to the control groups. High plasma levels of methadone were observed in individuals with the CYP2B6^*^5 homozygous genotype compared to the wild type and heterozygous genotype (Ahmad et al., 2017). Studies also show that CYP2B6 genotype influences the severity of neonatal abstinence syndrome (Mactier et al., 2017) in infants of methadone-maintained opioid-dependent mothers. Infants who needed treatment were more likely to carry the wild type genotype for CYP2B6^*^6 allele (516GG, 785AA) (Mactier et al., 2017). Studies involving Malay opioid dependent males revealed association between CYP2B6^*^6 and increased severity of pain (Zahari et al., 2016). The presence of concomitant diseases such as HCV infection influence methadone therapy. Reportedly, HCV patients often require higher doses of methadone. Most studies have reported the inability of patients, harboring the CYP2B6^*^6 allele to metabolize and clear the drug (Crettol et al., 2005; Hung et al., 2011; Bart et al., 2014; Csajka et al., 2016). Thus, patients are likely to experience unwanted effects of the drug. Therefore, dose reduction in this group of patients may yield methadone safety. Meanwhile, others have shown that the CYP2B6^*^4 allele results in increased metabolism of the drug, thus patients might require increased dose. Patient genotyping for CYP2B6 variants may be of importance when considering dose requirement in methadone maintenance treatment most especially among HCV and HIV patients.

Even though methadone maintenance therapy is effective and affordable, it is still unavailable in many LMICs where it is highly needed. High prevalence of HIV, hepatitis C virus (HCV) and tuberculosis is reported among people with drug use disorder (Wu and Clark, 2013; Larney et al., 2017). As reported in 2018, out of 179 countries with evidence of drug use disorder, opioid substitution therapy (OST) was available in only 86 countries (Avert, 2016; International HR, 2019). OST is a replacement therapy whereby prescribed medications such as methadone and buprenorphine are given to opioid dependent patients, which enables them to reduce or cease from injecting drugs. OST is not found in Nigeria and Zimbabwe despite the presence of drug addicts and HIV patients. However, the Nigerian government has initiated guidelines on the use of methadone for treatment of drug rehabilitation (International HR, 2018). OST is available in South Africa and on a smaller scale in Tanzania, Uganda, Senegal, and is highly expanding in Zanzibar and Kenya (International HR, 2019). In Asia, OST is present in most countries with the highest number in China and the least in Cambodia (International HR, 2019). In Eastern Europe and Central Asia, OST is applied in many countries but coverage is limited. In Western Europe and North American countries, OST is vastly available. However, in Germany opiate substitution treatment is variable between people in prison and those living outside of prison. According to a report by Stöver et al., the application of OST in German prisons depends on the federal state, the prison and prison doctors (Stöver et al., 2019). Existing barriers to accessing OST in both high and LMICs include criminalization and financial barrier, for example, OST is forbidden in Russia and Uzbekistan. Though OST is free in Australia, most people still buy it at a minimum cost of AU35 per week. If people with opiate addiction are not treated, the prevalence of HIV will continue to rise in LMICs. There is a need for more countries to provide OST with good coverage so as to reduce HIV, hepatitis C and mortality among drug users. There is need for the government to provide funds for the health care in some LMICs.

### Artemisinin

Artemisinin-based combination therapy (ACT) is the basis of treatment and considered first-line for the majority of malaria infection cases (WHO, 2015). ACT, presently approved for treatment of uncomplicated *Plasmodium falciparum* malaria in many malaria-endemic countries, are substrates of CYP enzymes (Svensson and Ashton, 1999). Combining artemisinin agents, which are fast acting with a short half-life with partner drugs that have long half-life enables optimization of parasite killing and greatly protects against reinfection (White et al., 2014). Patient's response to antimalarial treatment can be influenced by factors such as quality of the antimalarial agent, the natural immune system of the host, parasite resistance, concomitant diseases and the pharmacokinetics of the malaria drug (Travassos and Laufer, 2009). Most studies on the efficacy of antimalarial agents have focused more on understanding the resistant mechanism of the parasite by investigating the parasites multi drug resistant genes (Travassos and Laufer, 2009). However, studies have shown interindividual variations in the concentration of artimisinin, dihydroartemisinin, artesunate, and their anti-malarial effect among malaria infected individuals. Underdosing seems to enhance parasite resistance to the therapeutic agents. Due to the lack of efficacy, optimization of antimalarial treatment is the main factor considered toward global eradication of the diseases (WHO, 2015).

Among many other factors, host genetics, especially polymorphisms in CYP enzymes involved in the metabolism of the drugs, may be one of the confounders, causing variability in ACT drugs levels and treatment failure. *In vitro* studies show that artemisinin metabolism in human liver microsomes is mediated primarily by CYP2B6 with a contribution of CYP3A4 in individuals with low expression of CYP2B6 (Svensson and Ashton, 1999). So far, a report from Tanzania indicated their concern about metabolism of ACT and high prevalence of CYP2B6 G516T and other CYP polymorphisms in the population. The prevalence of CYP2B6 G516T in the Tanzanian population was 36% (Marwa et al., 2014). Authors did not evaluate the direct impact of these polymorphisms on treatment outcome or safety of the drugs (Marwa et al., 2014). Another study in the malaria-endemic population of Timor Leste indicated that the prevalence of CYP2B6^*^4, ^*^9, and ^*^6 might impact the metabolism and efficacy of artemisinin and its derivatives among the Timorians (Hananta et al., 2018). Another study amongst Nigerian HIV-malaria infected subjects explored the impact of CYP2B6 516GT polymorphism on NVR and artemether-lumefantrine drug-drug interaction. The authors showed that decreased exposure to artemether and desbutyl-lumefantrine caused by NVR was further enhanced by patients with CYP2B6 516GG genotype (ultrarapid metabolizers) (Abdullahi et al., 2020). Again, the CYP2B6 516TT genotype (poor metabolizers) also influenced increased exposure to dihydroartemisinin and lumefantrine caused by NVR (Abdullahi et al., 2020). According to the authors, the inductive effect of NRV on CYP2B6 and CYP3A4 enzymes, both of which are involved in the metabolism of these antimalarial drugs, might have caused this variability (Abdullahi et al., 2020). An Iranian study also indicated high prevalence of CYP2B6^*^2, ^*^4, ^*^5, ^*^6, and ^*^7 alleles among the Iranian Baluchi, which may affect patient response to artemisinin and derivatives (Zakeri et al., 2014).

Limited effort has been employed to determine genetic polymorphisms in CYP enzymes, which may lead to therapeutic failure in patients who are extensive metabolizers or cause toxicity and resistance in patients who are slow metabolizers (Gil, 2013). ACT is an effective treatment for resistant malaria, knowledge on polymorphisms influencing their efficacy may help to improve malaria therapy. The fight against over the counter drugs will help to reduce relapse among patients and improve the lives of people in the rural population.

The quality of malaria care offered in many malaria pandemic LMICs is still very poor, presumption diagnosis is common whereby treatment is provided to patients without any malaria test inspite of the WHO recommendation of “test and treat” (Macarayan et al., 2020). Over the counter poor quality malaria drugs, some of which have no active ingredients, are offered especially by smaller vendors (Bassat et al., 2016; Walker et al., 2018). In Uganda, compared to the urban population, the rural populations spent more money and experienced 97.9% of deaths due to poor quality antimalarial drugs.

## Discussion and Future Perspective

### CYP2B6 Pharmacogenetics and Drug Response

This article provides evidence on CYP2B6 functional variability in drug metabolism and exposure across populations. The impact of CPY2B6 variant on patient response to various substrates is evident in most ethnicities involved in this study. Depending on CYP2B6 genotype, patients may be vulnerable to ADRs ranging from mild to severe due to increased exposure to active oral drugs, or otherwise experience therapeutic failure due to reduced exposure to active metabolite in the case of prodrugs. Poor metabolizers are likely to experience more ADRs (active compounds) or treatment failure (prodrugs) than intermediate or normal metabolizers. According to literature, CYP2B6^*^6, which is a haplotype of two variants, CYP2B6^*^4 and CYP2B6^*^9, is the most studied allele (Li et al., 2012; Desta et al., 2021). Due to the increasing knowledge on the role of CYP2B6 enzyme in drug metabolism, there is the need to evaluate other CYP2B6 variants on substrate metabolism in various populations or ethnicity. According to the information gathered, some studies considered the G516T (^*^4) variant as CYP2B6^*^6 allele, to maintain a common style of variant nomenclature in articles. Researchers can make use of the publicly available pharmacogene variation (PharmVar) website, which clearly defines each haplotype or alleles (Desta et al., 2021). CYP2B6 loss of function alleles (poor metabolizer genotypes), which lead to an increase in individual active drug exposure and toxicity are frequent in some populations leading to high risk of ADRs in these populations.

In order to achieve the Joint United Nations Programme on HIV/AIDS (UNAIDS) 90-90-90 target and complete eradication of AIDS by 2030, pharmacogenetics testing for CYP2B6 to assist EFV therapy in patients who are unlikely to benefit from dolutegravir is of urgent need (Masimirembwa et al., 2016; Mukonzo et al., 2016). The Clinical Pharmacogenetics Implementation Consortium guideline for CYP2B6 and EFV-containing antiretroviral therapy might serve as a basis for implementation of CYP2B6 pharmacogenetic testing for EVF therapy (Desta et al., 2019). This may help to reduce ADRs and increase patient compliance with subsequent reduction in drug resistance due to lack of patient compliance. At the moment, many pharmacogenomics related ADRs are noticed only after the drugs have been administered to the patients. Population specific pharmacogenomics approaches at the level of drug development can be used to address differences in susceptibility to ADRs between populations. The inclusion of pharmacogenomics in clinical trials could give a clue on populations that are likely to benefit or suffer from adverse effects. This could guide dose adjustments for some populations. Previous clinical trials included individuals from African descent to represent the African population, while it is clear that the African continent demonstrates high genetic diversity. Likewise, Asian Americans or Asian Europeans cannot represent the Asian population. Therefore, the inclusion of a diverse population in clinical trials is inevitable. Interestingly, intrapopulation as well as inter-individual variability in CYP2B6 alleles further complicates the efficacy of many of its substrates. This could potentially be improved by the application of patient genotyping prior to prescription in clinical practice. Thus, a robust and more personalized therapy could be provided to the patients.

### Availability and Accessibility of Medication

According to our findings, HAART combination therapy that includes EFV or NVP is mostly used in LMICs despite the high frequency of CYP2B6 loss of function alleles in these populations. Thus, pharmacovigilance is urgently needed in these populations for the detection and subsequent prevention of ADRs. Contrary to LMICs, in HICs more potent, less toxic and novel antiretroviral drugs are used quite often. Higher donor dependency and cost of medication has been highlighted as barriers to the accessibility of quality and less toxic drugs in LMICs. Donor funding has saved many lives in LMICs for the past decades. The help from richer countries is provided in the form of finance or medication via Government officials or private agencies in health sectors. With the growing population in LMICs, it is evident that donor funding can no longer benefit every individual. Therefore, in this era, donors should focus more on human capacity building and establishment of infrastructures that will help LMICs become independent or self-sponsored (Pillai et al., 2018). For example, antiretroviral drugs are manufactured and sold at a cheaper rate in India compared to African countries, where drugs are mostly imported at a very high cost (Dickson, 2001). The African pharmaceutical industry, for example, could be expanded and strengthened. This could help to remove financial barriers to medicines as well as to improve access to more potent, expensive, less toxic medication. The public health sector needs to fully support organizations such as the African Pharmacogenomics Consortium (APC), which seeks to address the issue of drug safety, financial problems in the health care sector, disease burden, research training and implementation of pharmacogenomics in Africa (Dandara et al., 2019). There is a need for creating awareness, funding and provision of medication in countries where mental health has been neglected. For example, a significant reduction of heroin use and improvement of mental health was observed among participants who were retained for methadone therapy for 6 months in South Africa (Scheibe et al., 2020).

### Health Polices and Patent

There is a need for amendments of foreign and government policies, that limit the growth of health care. According to Tomlinson et al., amending the patent law could improve affordability and accessibility of medicines in South Africa (Tomlinson et al., 2019). The government in LMICs needs to develop strategies to raise internal funds to support health care rather than solely depending on foreign aid. There is a need for allocation of healthcare budgets in both public and private sectors. Additionally, accountability and better amendment of funds in healthcare can help to improve health care services.

### Comorbidities

People with two or more diseases need special attention and health care coverage, for example, people with HCV and HIV coinfection. The treatment of HIV might worsen HCV infection in such individuals. Thus, increasing health coverage and accessibility to diagnosis and counseling by trained medical staffs can help patients to receive the right therapy and avoid drug-drug interactions due to concomitant use of HIV and HCV drugs.

## Conclusion

In conclusion, there is a high level of CYP2B6 genetic variability between and within ethnicities. In addition to other confounders that can affect the pharmacokinetics and pharmacodynamics properties of a drug, CYP2B6 genotyping could be considered in regards to all CYP2B6 substrates prescriptions in populations with expected high variability and drugs with narrow therapeutic window.

## Author Contributions

JS developed the presented idea, supervised the manuscript progress and data analysis, and published own data on CYP2B6 pharmacogenetics. SY, KJ, JB, and CM contributed to the manuscript content, data analysis, and literature search. IL did the systematic review of the literature, evaluated, analyzed the impact of the CYP2B6 alleles on drug safety in a global view, and wrote the manuscript. All authors contributed to the article and approved the submitted version.

## Conflict of Interest

The authors declare that the research was conducted in the absence of any commercial or financial relationships that could be construed as a potential conflict of interest.

## Footnotes

^1^Drugbank. Available online at: https://go.drugbank.com/drugs/DB00625.

^2^FDA. Available online at: https://www.accessdata.fda.gov/drugsatfda_docs/nda/2019/210649Orig1s000TOC.cfm.

^3^WHO. Available online at: https://digicollections.net/medicinedocs/#/d/s19018en.2010.

^4^WHO. *Tobacco Free Initiative*. Available online at: https://www.who.int/tobacco/mpower/en/.
